# Emerging Fatal Ib/CC12 Hypervirulent Multiresistant *Streptococcus agalactiae* in Young Infants With Bloodstream Infection in China

**DOI:** 10.3389/fmicb.2021.767803

**Published:** 2021-12-15

**Authors:** Jingxian Liu, Feng Chen, Hongyan Guan, Jiajia Yu, Jing Yu, Jing Zhao, Ying Liu, Lisong Shen

**Affiliations:** ^1^Department of Clinical Laboratory, Xin Hua Hospital, Shanghai Jiao Tong University School of Medicine, Shanghai, China; ^2^Faculty of Medical Laboratory Sciences, Shanghai Jiao Tong University School of Medicine, Shanghai, China

**Keywords:** *Streptococcus agalactiae*, bloodstream infection, molecular epidemiology, virulence, antibiotic resistance

## Abstract

*Streptococcus agalactiae* [also known as group B *Streptococcus* (GBS)] is a tremendous threat to young infants. Eighty pediatric GBS infection cases were enrolled from a teaching hospital in Shanghai between 2009 and 2020; among them, 72.5% (58/80) were diagnosed with bloodstream infection (BSI). Sequence types (STs) and serotypes of associated GBS strains were identified, and most of the Ib/clonal complex (CC)12 (86.7%, 13/15) strains caused BSIs, which was significantly higher than that of the genetically related clone Ib/CC10 (20%, 2/10; *p* < 0.05). Ib/CC12 BSI (30.8%) mortality was significantly higher than that of non-Ib/CC12 BSI (2.2%; *p* < 0.05). Virulence genes associated with adhesion, invasion, and immune evasion were detected using polymerase chain reaction. The *fbsA* and *gbsPC1* positive rates of Ib/CC12 strains was higher than that of non-Ib/CC12 strains, whereas *cpsIaJ*, *cpsJ*, *cpsI*, and *cpsG* positive rates were lower than those of non-Ib/CC12 (*p* < 0.05). In *in vitro* studies, the Ib/CC12 strains had strong invasiveness in RAW264.7 cells, but less invasiveness in human umbilical vein endothelial cells, human brain microvascular endothelial cells, and human mammary epithelial cells when compared to other two clones. In the *in vivo* model, the Ib/CC12 GBS invaded the circulation system more rapidly after intraperitoneal injection, was more difficult to eradicate by phagocytes, and caused significantly higher mortality than Ib/CC10 and III/ST17 (*p* < 0.05). Genome analysis showed that the Ib/CC12 strains had two clustered regularly interspaced short palindromic repeat-Cas systems and carried more antibiotic resistant genes, which conferred resistance to macrolides, clindamycin, aminoglycosides, and tetracycline. The Ib/CC12 strains had 45 unique annotated genes compared to that of Ib/CC10, including the pathogen-related toxin/antitoxin system, PezA/T. In conclusion, Ib/CC12 is an emerging hypervirulent multiresistant GBS clone that causes invasive and fatal infections in pediatric patients. The prevention and control of Ib/CC12 GBS infection should be emphasized.

## Introduction

*Streptococcus agalactiae* (also termed as group B *Streptococcus*, GBS) is a leading cause of invasive disease in neonatal and young infant patients, although intrapartum antimicrobial prophylaxis (IAP) has significantly reduced the incidence of early onset GBS infection (Tan et al., 2021; Vatne et al., 2021; Wong et al., 2021). A recent survey revealed that families with a baby with GBS infection suffered significant financial and psychological difficulties (Raybould et al., 2021). Invasive infection in young infant patients caused by GBS is often very serious, and the overall case fatality rates range from 1–8.4% in term infants to 5–20% in preterm infants (Raabe and Shane, 2019). The leading syndromes of invasive GBS infections are sepsis and meningitis; meningitis often occurs as a complication of GBS sepsis, since GBS crosses the blood–brain barrier through transcellular penetration when bacterial density in the bloodstream increases to a certain level (Zhu et al., 2021). Infants who survive the acute phase of GBS meningitis infection may have significant cognitive or neurological sequelae; 32–44% of infants with GBS meningitis have neurodevelopmental impairment, with severe impairment occurring in up to 19% of surviving infants (Libster et al., 2012; Kohli-Lynch et al., 2017). Formally, sequence type 1 (ST1) GBS is associated with intrauterine fetal death, and III/ST17 is considered a hypervirulent clone that is prevalent worldwide and is associated with sepsis and meningitis, especially in GBS early onset disease (Schindler et al., 2020). However, GBS epidemiology in pediatric patients is changing in the post-IAP era; the number of other GBS clones isolated from pediatric invasive disease cases are increasing (Zhang et al., 2021). As antibiotic resistance increases (Hayes et al., 2020; Mudzana et al., 2021) and a vaccine is clinically unavailable (Berner, 2021), prevention of prenatal GBS remains a significant challenge. Currently, there are debates on IAP in considering antibiotic abuse and negative effects on the intestinal microbiota of newborns (Walker et al., 2021). Thus, it is necessary to determine the clinical and molecular features of GBS isolated from clinical samples to optimize GBS prevention and treatment strategies.

Therefore, this study was conducted to investigate and characterize the ST, serotype, and virulence of GBS isolated from pediatric patients and analyze the clinical features of GBS bloodstream infection (BSI) cases. Ib/clonal complex (CC)12 GBS that caused BSI had significantly higher mortality than that of non-Ib/CC12 GBS BSI, suggesting that it was an emerging hypervirulent clone. To investigate the pathogenicity and pathogenic mechanism of Ib/CC12, internalization features were tested using different cell lines, and the pathogenic process was determined using a C57BL/6 mouse infection model. Whole genome sequencing was used to investigate the genetic background of Ib/CC12, and predict potential virulence determinants that might be responsible for the lethal clinical features of this clone.

## Materials and Methods

### Study Design

This was a 12-year retrospective observational study conducted at Xinhua Hospital affiliated with Shanghai Jiao Tong University School of Medicine, a 3,000-bed tertiary care university teaching hospital in Shanghai, China. Pediatric patients (≤ 1 years old) diagnosed with GBS infection between January 2009 and December 2020 were included in the study. Patients with no GBS isolates collected or preserved were excluded. All medical records of the enrolled patients were reviewed by a team of physicians from the Microbiology Department and the Infectious Diseases Department. The following variables were collected from medical charts: demographic characteristics (age, sex, admission year, and admission ward), birth status (preterm birth and cesarean delivery), maternal history (maternal age, gravidity, and parity), comorbidities (pneumonia, meningitis, and urinary tract infection), and biomarker levels (leukocyte counts, platelet counts, hemoglobin, procalcitonin, C-reactive protein, albumin, total bilirubin, creatinine, and lactate). The severity of organ failure was measured using the Sequential Organ Failure Assessment (SOFA) score adapted for children (Matics and Sanchez-Pinto, 2017). Biomarker levels and risk scores at the time of GBS-BSI onset were determined within 24 h after the index blood culture.

### Isolate Collection and Identification

GBS strains isolated from clinical samples were stored at −80°C in glycerin broth. Isolates were then recovered and cultured on 5% sheep blood plates for 24 h at 37°C in a 5% CO_2_ atmosphere, and then re-identified using matrix-assisted laser desorption/ionization-time of flight mass spectrometry (MALDI-TOF MS; Microflex^™^ LT, Bruker Daltonik, Germany). The inclusion criterion was patients aged ≤1 years; for patients with bloodstream infection, the first strain isolated from blood culture was enrolled, while for non-bloodstream infection patients, the first strain isolated from a clinical sample of the infection site was enrolled.

### Serotyping

The nine GBS serotypes (Ia, Ib, II–VII) based on capsular polysaccharide (CPS) were distinguished using previously developed multiplex polymerase chain reaction (PCR) methods (Poyart et al., 2007).

### Multilocus Sequence Typing and Homology

Multilocus sequence typing was performed by sequencing seven housekeeping genes *adhP*, *pheS*, *atr*, *glnA*, *sdhA*, *glcK,* and *tkt,* as previously described (Jones et al., 2003). The ST was determined using the *S. agalactiae* MLST database.^1^ New alleles or ST profiles were submitted and assigned to the *S. agalactiae* MLST database. Bionumeric 8.0 was used for homology analysis.

### Virulence Genes

Forty-five virulence genes associated with adhesion, invasion, and immune evasion were detected using PCR. Primers and amplification conditions have been previously described (Jiang et al., 2016). PCR products were visualized using agarose gel electrophoresis and the SYBR safe gel stain.

### Cell Invasion Assay

Human umbilical vein endothelial cells (hUVECs), human brain microvascular endothelial cells (hBMEC), human mammary epithelial cells (MCF-10A), and murine macrophages (RAW264.7) were used in the cell invasion assay. hUVEC, hBMEC, and RAW264.7 were cultured in Dulbecco’s modified Eagle medium (DMEM; Thermo Fisher Scientific, Grand Isaland, NY, United States), supplemented with 10% fetal bovine serum (FBS; Gibco-Invitrogen Corp., Carlsbad, CA, United States) and 100 mg/ml gentamicin and 100 U/ml penicillin. MCF-10A cells were cultured in mammary epithelial cell medium (ScienCell, Carlsbad, CA, United States), supplemented with matched mammary epithelial cell growth supplement (ScienCell, Carlsbad, CA, United States), penicillin/streptocmycin solution (Sciencell, Carlsbad, CA, United States), and 10% FBS. Cells were incubated at 37°C in a 5% CO_2_ atmosphere. GBS internalization by cells was assessed using an antibiotic protection assay (Ogawa et al., 2011). Briefly, cultures were grown in Todd-Hewitt broth and harvested at the mid-exponential phase of growth by centrifugation for 10 min at 3000 × *g*, washed once by 1 × PBS, and resuspended in DMEM (hUVEC, hBMEC, and RAW264.7) or DMEM/F12 (MCF-10A). Cells were infected with GBS strains at a multiplicity of infection of 1:100 for 2 h. Cells were washed twice with phosphate-buffered saline (PBS) and then incubated with DMEM or DMEM/F12 supplemented with lysozyme (1 mg/ml) and penicillin (5 mg/ml) for an additional 2 h. Cells were then washed twice with 1 × PBS, lysed using sterilized water, and bacterial numbers were counted following inoculation onto Brain Heart Infusion (BHI) agar plates. Bacterial density was confirmed by plate counts.

### Infection Model

Male or female C57BL/6 mice (8–12weeks old) were purchased from the Jihui Laboratory Animal Breeding Limited Company (Shanghai, China). C57BL/6 mice were infected intraperitoneally with 0.1 ml PBS containing GBS 1 × 10^7^ cells. For survival analysis, three strains were randomly selected from each CC (CC12 and CC17 in the BSI group and CC10 in the non-BSI group), and the results of three duplicate independent experiments were pooled. The observation endpoint was death or 1week after infection. For bloodstream bacterial load detection, 5 μl venous blood was collected from the tail of mice each hour after infection, then diluted and inoculated onto BHI agar for bacterial density counting. For pathological studies, mice were euthanized 12 h after infection. Peritoneal macrophages were obtained, as previously described (Salazar et al., 2019). Briefly, a midline incision was made, followed by lavage of the abdominal cavity using 2 ml cold, sterile PBS. The peritoneal fluid containing macrophages was used to directly make smears, then fixed and stained using the Gram stain method. Organs, including the brain, spleen, liver, and kidney were collected and formalin-fixed and paraffin-embedded. Pathological sections were stained using hematoxylin and eosin and viewed using an optical microscope.

### Whole Genome Sequencing

Three isolates were selected from the CC12 (termed B1, B5, and B7) and CC10 (termed N1, N2, and N3) GBS strains, respectively. Total DNA was extracted using the lysozyme–sodium dodecyl sulfate–proteinase K method. Whole genome sequencing was performed using the Illumina HiSeq2500 system (Illumina, San Diego, CA, United States). SPAdes (version 3.9.0) was used to assemble the sequences. Protein function annotation was performed using the Basic Local Alignment Search Tool (version 2.2.31+) and HMMER (version 3.1b1). OrthoVenn 2.0 was used for orthologous cluster analysis. The CRISPR recognition tool (version 1.2) was used to identify clustered regularly interspaced short palindromic repeats. The assembled draft genomes of the isolates were submitted to NCBI genome.

### Statistical Analysis

Continuous variables were presented as the mean ± standard deviation (SD) or as medians with an interquartile range. Categorical variables were described as frequencies. Continuous variables among groups were compared using Student’s *t*-test and the nonparametric Mann–Whitney *U*-test. Proportions were compared using a *χ*-squared test or Fisher’s exact test. Survival curves were plotted using the Kaplan–Meier method and differences in survival were calculated using the log-rank test. Differences were considered significant at *p* < 0.05. GraphPad Prism 8.0 and R software were used for all statistical analyses.

### Ethics Statement

This study was approved by the ethics committee of Xinhua Hospital, Shanghai, People’s Republic of China, and all experiments were conducted in accordance with animal protocols approved by the Animal Care and Use Committee at Xinhua Hospital (XHEC-C-2020-080).

## Results

### Study Population and GBS Isolate Characteristics

From 2009 to 2020, a total of 613 GBS infectious disease cases were identified, including 125 pediatric patients and 488 adult patients. For young infant patients ≤1 year old, 80 GBS isolates were preserved and enrolled for further study. Among the 80 patients, 72.5% (58/80) had BSI or BSI complicated with other site infections, such as meningitis, pneumonia, or urinary tract infection, whereas 27.5% (22/80) had a single-site infection that did not progress into a BSI or other severe invasive infections. The later included 20% (16/80) with urinary tract infection, 2.5% (2/80) with soft tissue infection, 3.75% (3/80) with pneumonia, and 1.25% (1/80) with entophthalmia.

**Figure 1:**
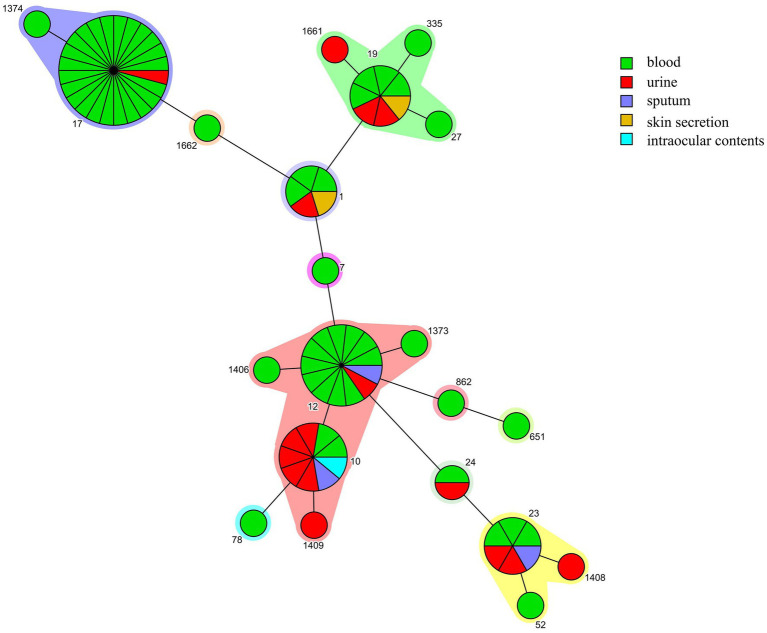
Minimum spanning trees of group B *Streptococcus* (GBS) strains. Each node represents a single sequence type (ST). The node size is proportional to the number of isolates within the represented ST. The distance of the node represented the relationship between STs. The origin of GBS strains isolated from different samples are depicted with different color.

All associated 80 GBS isolates were re-identified as GBS using MALDI-TOF MS. A total of seven serotypes were detected in the 80 strains, serotype III (46.25%, 37/80) was the most common, followed by Ib (32.5%, 26/80) and Ia (10.0%, 8/80). The 80 strains were divided into 21 STs (14 were previously described STs and 7 were novel STs), with ST17 (30.0%, 24/80) being the most prevalent clone, followed by ST12 (16.25, 13/80) and ST10 (11.25, 9/80). The serotypes were correlated with CC, with all of the CC17 and 80.0% (8/10) of the CC19 strains belonging to serotype III, all the CC12 and CC10 strains were serotype Ib, and 87.5% (7/8) of the CC23 strains were serotype Ia. III/CC17 (41.4%, 24/58) was the most common clone causing BSI, followed by Ib/CC12 (22.4%, 13/58) (Table 1). Interestingly, 86.7% (13/15) of the Ib/CC12 strains caused BSI, which was not significantly different when compared to the hypervirulent clone III/CC17 (96.0%, 24/25; *p* = 0.64). CC10 and CC12 were genetically related since their founders ST10 and ST12, respectively, were single locus variants (SLVs) with only one different allele. However, only 20.0% (2/10) of the CC10 strains caused BSI (Figure 1), which was significantly lower than CC12 (*p* < 0.05).

**Table 1:** Characteristic of GBS in different CCs.

	CC1	CC10	CC12	CC17	CC19	CC23	Others
Isolates, n	5	10	15	25	10	8	7
STs (n)	ST1 (5)	ST10 (9), ST1409 (1)	ST12(13), ST1373(1), ST1406(1)	ST17(24), ST1374 (1)	ST19 (7), ST27(1), ST335(1), ST1661(1)	ST23(6), ST52(1), ST1408(1)	ST24(2), ST7(1), SST78(1), ST651(1) ST862(1), ST1662(1)
Serotypes (n)	III(1), V(2), VI(1), VII (1)	Ib (10)	Ib (15)	III(25)	III(8), V(1),VII (1)	Ia (7), ND (1)	Ia (1), Ib (1), III(3), V(2)
Origin (n)	Blood(3), urine(1), skin secretion(1)	Blood(2), urine(6), sputum(1), intraocular contents (1)	Blood(13), urine(1), sputum(1)	Blood(24), urine(1)	Blood(6), urine(3), skin secretion(1)	Blood(4), urine(3), sputum (1)	Blood (6), urine (1)
Onset of disease	EOD (2), LOD (2), LLOD (1)	EOD (2), LOD (8)	EOD (4), LOD (10), LLOD (1)	EOD (4), LOD (21)	EOD (5), LOD (3), LLOD (2)	EOD (3), LOD (3), LLOD (2)	EOD (3), LOD (4)

*CC, clonal complex; ST, sequence type; EOD, early onset disease; LOD, late onset disease; LLOD, late late onset disease*.

### Ib/CC12 GBS BSI Clinical Features

For the CC12 GBS BSI cases, 69.2% of them occurred in males, which was significantly higher than that of non-CC12 GBS cases (*p* < 0.05). Most of the patients were full-term infants with normal weight. Although not statistically different, patients with CC12 GBS BSI had a higher rate (76.9%) of concurrent infections than those with non-CC12 GBS BSI (57.8%), and the most common concurrent infection was meningitis. Approximately 69.2% of CC12 GBS BSI cases were late-onset disease (LOD). The average procalcitonin level in patients with CC12 GBS BSI was higher than that of patients infected with non-CC12, but the difference was not statistically different (*p* = 0.164). Notably, the SOFA score and patient mortality with CC12 BSI were significantly higher than those of patients infected with non-CC12 BSI (*p* < 0.05) (Table 2).

The overall GBS BSI mortality rate was 8.6% (5/58); among the five death cases, four of them were caused by Ib/CC12 and one by the VII/CC1 strain (Table 2). All cases had LOD with no abnormal condition after birth, except for one patient, who had bronchial pneumonia, but he recovered well before the onset of GBS BSI. All of these cases had a hidden onset, but rapidly progressed. The patients were admitted to the hospital and received proper treatment on time; however, the outcome was horrendous. All patients died within 1week from the time when symptoms occurred and the shortest time was 18 h.

**Table 2:** Different characteristics between CC12 and non-CC12 GBS causing BSI.

Variable	CC12 (N = 13)	Non-CC12 (N = 45)	*p* value
Boys, n (%)^*^	9 (69.2)	16 (35.6)	0.031
Premature, n (%)	2 (16.7)	3 (6.8)	0.625
Low birth weight, n (%)	2 (16.7)	3 (6.8)	0.625
Birth weight (g)	3,076 ± 600	3,167 ± 680	0.675
Number of infection sites	2 (2–2)	2 (1–2)	0.387
Complicated with meningitis	7 (53.8)	18 (40.0)	0.375
Onset age (d)	12 (1,20)	13 (1,25)	0.97
LOD	9 (69.2)	26 (57.8)	0.457
ICU stay	3 (23.1)	8(17.8)	0.978
Mother history			
Maternal age	29 (28–32)	29 (27–32)	0.581
Gravidity, median (IQR)	1 (1,1.75)	1 (1,2)	0.7647
Parity, median (IQR)	1 (1,1)	1 (1,1)	0.9341
Cesarean delivery, n (%)	8 (66.7)	26 (57.8)	0.821
Laboratory examinations			
WBC (×10^9^/L)	10.1 ± 9.9	10.8 ± 6.2	0.751
Hb (g/L)	138.8 ± 30.2	139.9 ± 27.0	0.898
N %	62.1 ± 17.1	59.1 ± 16.2	0.564
PLT (×10^9^/L)	259.5 ± 168.7	304.3 ± 107.3	0.252
CRP (mg/L)	28.3 ± 29.7	28.1 ± 39.5	0.985
PCT (ng/ml)	47.2 ± 48.6	27.5 ± 35.6	0.146
TP (g/L)	53.2 ± 5.1	55.1 ± 6.8	0.363
Albumin (g/L)	33.4 ± 3.7	33.7 ± 4.1	0.843
TBIL (μmol/L)	91.0 ± 66.5	98.9 ± 68.0	0.71
Creatinine	41.138 ± 24.245	35.871 ± 13.400	0.310
Lactic acid	4.258 ± 2.856	3.131 ± 2.162	0.145
SOFA score (IQR)^*^	4 (3,8)	3 (2,3)	< 0.01
Length of hospital stay	14 (5–33)	15 (8–24)	0.526
Outcome^*^			0.01
Died, n (%)	4 (30.8)	1 (2.2)	
Relapsed, n (%)	1 (7.7)	0 (0.0)	
Recovered, n (%)	8 (61.5)	41 (91.1)	

GBS, group B Streptococcus; BSI, bloodstream infection; ICU, intensive care unit; IQR, interquartile range, LOD, late-onset disease; WBC, white blood cell count; Hb, hemoglobin; N, neutrophil; PLT, platelet counts; CRP, C-reactive protein; PCT, procalcitonin; TP, total protein; TBIL, total bilirubin; and SOFA, Sequential Organ Failure Assessment. ^*^P < 0.05, the difference was significantly different.

### Virulence Genes of Ib/CC12 GBS Isolates

All 80 GBS strains were positive for the virulence genes fibrinogen binding protein B (*fbsB*), cytolysin X (*cylX*), *cylD, cylG*, acyl carrier protein C (*acpC*), *cylZ*, *cylA*, *cylB*, *cylE*, *cylF*, *cylI*, hyaluronate lyase B (*hylB*), c alpha protein (*bca*), UDP-N-acetylglucosamine 2-epimerase A (*neuA*), *neuC* and penicillin-binding protein 1A (*pbp1A*). The positive rates of *fbsA*, the major subunit protein of the pilus cluster (*gbsPC1*), cell surface protein (*spb1*), capsular polysaccharide IaJ (*cpsIaJ*), *cpsJ*, *cpsI*, and *cpsG* genes were significantly different between CC12 and the other strains (Table 3). Virulence gene profiles were quite different among CCs. The virulence gene profile of CC12 was similar to that of CC10 and CC1, but CC12 was distinguished from CC1 by *fbsA* and distinguished from CC10 by *gbsPC1* (Supplementary Figure S1).

**Table 3:** Positive rate of virulence genes of CC12 and non-CC12 GBS strains.

Virulence genes	CC12 n, %	Non-CC12 n, %	*P* value
*fbsA* ^*^	15 (100.0)	22 (33.8)	< 0.01
*pavA*	14 (93.3)	65 (100.0)	0.420
*scpB*	15 (100.0)	62 (95.4)	0.925
*lmb*	15 (100.0)	60 (92.3)	0.605
*gbsPC1* ^*^	13 (86.7)	25 (38.5)	< 0.01
*gbsPC2*	13 (86.7)	36 (55.4)	0.051
*gbsPC3*	13 (86.7)	36 (55.4)	0.051
*gbsPC4*	13 (86.7)	36 (55.4)	0.051
*gbsPC5*	13 (86.7)	36 (55.4)	0.051
*cylJ*	15 (100.0)	62 (95.4)	0.925
*cylK*	0 (0.0)	10 (15.4)	0.234
*spb1* ^*^	15 (100.0)	15 (23.1)	< 0.01
*rib*	0 (0.0)	2 (3.1)	0.819
*bac*	1 (6.7)	12 (18.5)	0.467
*cpsM*	14 (93.3)	58 (89.2)	1.000
*cpsIaJ* ^*^	0 (0.0)	45 (69.2)	< 0.01
*cpsJ* ^*^	0 (0.0)	36 (55.4)	< 0.01
*cpsI* ^*^	0 (0.0)	37 (56.9)	< 0.01
*cpsG* ^*^	0 (0.0)	37 (56.9)	< 0.01
*cpsF*	14 (93.3)	63 (96.9)	0.925
*cpsE*	14 (93.3)	62 (95.4)	0.742
*cpsD*	15 (100.0)	64 (98.5)	0.420
*cpsC*	15 (100.0)	64 (98.5)	0.420
*cpsB*	15 (100.0)	64 (98.5)	0.420
*cpsA*	14 (93.3)	64 (98.5)	0.819
*neuD*	14 (93.3)	64 (98.5)	0.819
*neuB*	14 (93.3)	63 (96.9)	0.925
*cspA*	14 (93.3)	65 (100.0)	0.420

^*^P < 0.05, the difference is significant. GBS, group B Streptococcus.

### Ib/CC12 GBS Invasion Ability

**Figure 2:**
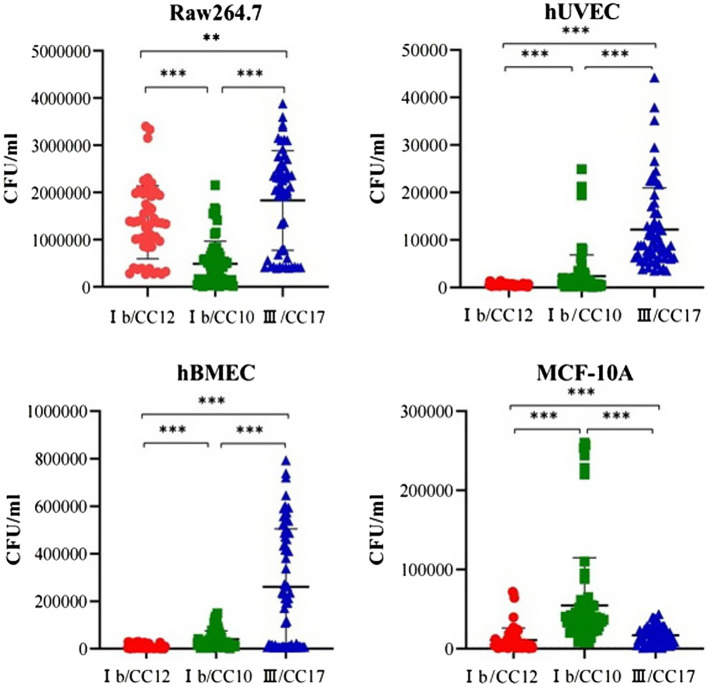
Group B *Streptococcus* (GBS) invasiveness towards different cell lines. The invasiveness of CC17 strains to human umbilical vein endothelial cells (hUVECs), human brain microvascular endothelial cells (hBMEC) and the murine macrophage cell line RAW264.7 was highest among the three clones. The invasiveness of CC12 strains to hUVECs, hBMEC and human mammary epithelial (MCF-10A) cells is significantly lower than those of CC10 strains. However, CC12 strain invasiveness to the RAW264.7 is significantly higher than that of the CC10 strains. ^**^ *p* < 0.01, ^***^ *p* < 0.0001.

Seven CC12 and seven CC17 strains from the BSI group, eight CC10 strains from the non-BSI group were randomly selected for a cell invasion assay. Intracellular survival was expressed as colony forming units/mL, which reflected a significant strain-dependent difference in invasiveness. All the strains were easier to invasive into RAW264.7, but harder to hUVEC. The CC10 strains was 3 and 5 times higher invasive to MCF-10A than CC17 and CC12, respectively. The invasiveness of CC17 strains was the highest in hUVEC, hBMEC and RAW264.7 cells. The CC12 strains showed no advantage in invading into hUVEC, hBMEC, and MCF-10A cells, but was significantly more invasive in RAW264.7 cells compared to that of CC10 strains (*p* < 0.05) (Figure 2).

### Ib/CC12 GBS Pathological Features

**Figure 3:**
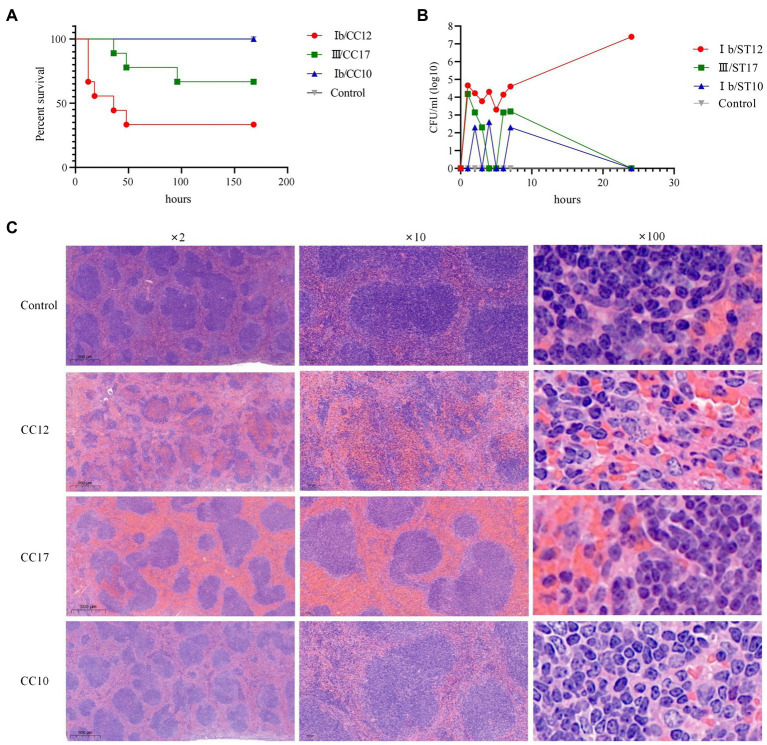
Pathological features of CC12 strains. **(A)** Kaplan–Meier curves, the survival rate of mice was significant lower after infected with CC12 than other GBS strains; **(B)** Bacterial density in bloodstream after infection, the CC12 and CC17 strains could invade into circulation system rapidly, but CC12 was harder to be eradicated; **(C)** Pathological section of the spleen, congestion could observed in both CC12 and CC17 infected mice, but the lymphonodules structure disruption, pyosis and gram-positive bacteria phagocytose (arrow point) were only observed in spleen section of CC12 infected mice.

Most mice (6/9, 66.7%) died within 60 h after infection with CC12, 33.3% (3/9) mice died within 100 h after infection with CC17, while all mice survived in the CC10 and control groups. Kaplan–Meier curves revealed that C57BL/6 mice with CC12 GBS infection had significantly higher all-cause mortality than those infected with CC10 and CC17 (Gehan-Breslow-Wilcoxon test, *p* < 0.05) (Figure 3A). The bacterial load at each time point showed that CC12 and CC17 invaded the bloodstream within 1 h after intraperitoneal administration. Bacterial load of CC17 in bloodstream decreased significantly 4 h after infection. However, the decreasing tendency of bacteria of CC12 in the bloodstream was moderate and the bacteria grew exponentially after 6 h (the experiment was performed at least twice with similar results and results from one experiment are shown in Figure 3B). Mice were euthanized after infection and their organs were collected. Compared to other groups, both CC12 and CC17 strain-infected mice was congested, but the most distinguishing feature of CC12 strain-infected mice was that the structure of splenic lymphonodules was damaged and gram-positive bacteria invading immune cells were observed in the pathological section (Figure 3C). The smear of peritoneal macrophages extracted from CC12-infected mice revealed that the CC12 strain invaded phagocytes, including neutrophils and macrophages, and lysed phagocyte cells that then released GBS (Supplementary Figure S2).

### Ib/CC12 GBS Genome Analysis

The CC12 genome size was slightly larger than that of CC10; a total of 2,275 ± 95 genes were predicted in the genome of CC12 strains, and 2,134 ± 9 in the genome of CC10 strains. All GBS strains shared 1922 orthologous clusters. There were 175 orthologous clusters found only in CC12 strains, among which 45 specific genes had annotations, including transposon elements (such as Tn3 and Tn916), agglutinin receptor precursor, CRISPR-associated protein, and toxin/antitoxin PezA/T. A total of 77 orthologous clusters were found only in CC10 strains, and 25 specific genes were annotated, mainly glycosyltransferase-and transcription-regulating associated proteins (Supplementary Figure S3). There were two CRISPR systems in CC12 strains, including type II-A and I-C, but only type II-A was observed in CC10 strains. The direct repeat (DR) was distinguishable between the different STs (Supplementary Figure S4).

Using virulence factor database analysis, 30 virulence factor genes were identified in the genome of CC12 strains and 28 in CC10 strains. The virulence profile was similar between the two clones, except that the ST10 strains lacked the *gbsPC1* (*gbs0628*) and *fbsA* genes.

Alignment with the Antibiotic Resistance Genes Database led to the identification of numerous resistance-associated genes in the genomes of CC12 strains, including the macrolide resistance determinant *mel* and *mefA* genes, clindamycin resistance determinants *lnuB* and *linB* (Mudzana et al., 2021; Swetha et al., 2021), tetracycline resistance determinant *tetO*, aminoglycoside resistance determinant *APH(3′)-IIIa*, *ANT(6)-Ia* and *ANT(6)-Ib*, streptothricin resistance determinant *sat-4*, cationic peptides resistance determinant *mprF*, pleuromutilin, lincosamide, and streptogramin A resistance determinant *lsaE*, and *ermB* genes, which exhibit the macrolide-lincosamide-streptogramin B phenotype (Table 4). However, only two resistance genes, *ermB* and *mprF,* were predicted in the genomes of the CC10 strains.

**Table 4:** Antibiotic resistant determinants in CC12 and CC10 strains.

Gene	CARD annotation	Function	CC12 (B1,B5,B7)	CC10 (N1,N2,N3)
*mel*	Putative ABC transporter ATP-binding protein YheS	Macrolide resistance	●○○	○○○
*APH(3')-IIIa*	Aminoglycoside 3'-phosphotransferase	Aminoglycoside resistance	●●●	○○○
*ErmB*	rRNA adenine N-6-methyltransferase	MLSb (macrolides-lincosamides-streptogramin B complex) phenotype	●●●	●●●
*ANT(6)-Ib*	Aminoglycoside 6-adenylyltransferase	Aminoglycoside resistance	●●●	○○○
*lsaE*	Putative ABC transporter ATP-binding protein YheS	Pleuromutilin, lincosamide, and streptogramin A resistance	●○○	○○○
*lnuB*	Hypothetical protein	Clindamycin resistance	●○○	○○○
*Streptococcus agalactiae mprF*	Phosphatidylglycerol lysyltransferase	Cationic peptides	●●●	●●●
*linB*	Hypothetical protein	Clindamycin resistance	●○○	○○○
*tetO*	Tetracycline resistance protein TetO	Tetracycline resistance	●●●	○○○
*sat-4*	Hypothetical protein	Streptothricin resistance	●●●	○○○
*mefA*	Enterobactin Exporter EntS	Erythromycin resistance	●○○	○○○
*ANT(6)-Ia*	Aminoglycoside 6-adenylyltransferase	Aminoglycoside resistance	○●●	

*CARD, The Comprehensive Antibiotic Resistance Database. ●, positive. ○ negative*.

## Discussion

*Streptococcus agalactiae* is a β-hemolytic, gram-positive bacterium, which is one of the most common pathogens causing invasive infections, such as BSIs and meningitis in young children (Madrid et al., 2017). Previously, III/CC17 GBS was considered a highly virulent clone, since it is a major cause of invasive neonatal disease (Poyart et al., 2008). In this study, III/CC17 strains also caused BSIs. However, it is worth noting that no matter in clinical cases or infection model in this study, Ib/CC12 appeared to be more fatal than III/CC17. The detection rate of Ib/CC12 in BSI was second only to III/CC17. The Ib/CC12 strains had a tendency to cause BSI as well as III/CC17, as the overwhelming majority of the Ib/CC12 and III/CC17 strains were isolated from BSI cases (*p* = 0.64). In addition, the outcome of Ib/CC12 GBS causing BSI was worse than that of other GBS clones (including III/CC17), with a mortality rate of 30.8%, which was also much higher than the reported average of the GBS-associated neonatal sepsis mortality rate of 10% (NICE Guideline Updates Team, 2021).

CC12 GBS has a higher vaginal tract colonization rate in Asian pregnant women than in other races, and recently, some studies have found that CC12 commonly causes invasive disease in adults, but has never been considered to be a prevalent clone in invasive disease in pediatric patients (Tien et al., 2011; Lee et al., 2019). In fact, the morbidity of CC12 GBS caused by pediatric invasive infection has been increasing gradually in the post-IAP area, but has not received sufficient attention (Li et al., 2019). To our knowledge, this is the first report of the prevalence of CC12 GBS causing invasive and fatal infections in pediatric patients. Of the five deaths in this study, four were caused by CC12 GBS and one was caused by CC1 GBS. Interestingly, the CC1 and CC12 strains shared similar virulence gene profiles, indicating that these strains might cause invasive disease through the same pathogenic mechanism. All five deaths were LOD, with a normal history of pregnancy and delivery, and no specific premonitory symptoms, but the condition of the patients deteriorated rapidly, even under sensitive antibiotic treatment. However, little is known about the virulence of the CC12 GBS. As Supplementary Figure S1 showed, the virulence gene profile of CC12 was quite different from that of the known hypervirulent clone CC17, for example, all the CC12 strains were positive for the *fbsA*, *spb1* genes but negative for *cpsIaJ*, *cpsI*, *cpsG* genes, while the detection rate was complete opposite in CC17 strains. Additionally, unlike CC17, the CC12 strains had no advantage in invasiveness of endothelial cell and epithelial cell, but it had a specific capability to invade RAW264.7 cells, a mouse splenic macrophage cell line. The invasion process to endothelial cell and epithelial cell would lead to strong inflammation response, the CC12 GBS infection certainly not conducted by this way. In the mouse infection model, the CC12 strains invaded the bloodstream rapidly but was eradicated hardly after being injected into the peritoneal cavity, and soon caused fatal infections. In addition, lysed phagocyte cells that released GBS cells were identified in peritoneal lavage fluids, and disruption of the spleen structure was observed in the pathological section of the spleen. Therefore, it was presumed that the CC12 pathological mechanism causes invasive infection mainly because it invades the immune system and interferes with its function. And it could explain why the onset of CC12 GBS infection was usually insidious but would develop seriously once the bacteria invade into bloodstream. However, much work remains to be done to verify this hypothesis.

As previously reported, CC10 and CC12 are related to serotype Ib (Furfaro et al., 2018). The founders of CC12 and CC10, namely ST12 and ST10 respectively, were SLVs. Genetically, CC12 was derived from CC10 and flourished as a sub-clone in evolution (Motallebirad et al., 2021). The genomes of CC12 and CC10 strains had more than 1700 orthologous clusters, but the genomes of CC12 were more unstable and variable because they had more mobile genetic elements. The CC12 strains also had more virulence genes and antibiotic resistance determinants than CC10 strains. The virulence gene profile was similar between CC12 and CC10, but the latter lacked *fbsA* and one pilus island gene *gbs0628*. Fibrinogen-binding protein *fbsA* is important for epithelial cell adherence, which plays a role in promoting bacterial adherence to host tissues during infection (Schubert et al., 2004). The pilus type of all the CC10 and CC12 strains was PI-1 + PI-2a; however, the CC10 strains lacked the major subunit protein of the PI-1 encoding gene *gbs0628*. PI-1 pili inhibit both GBS uptake and intracellular killing by macrophages (Jiang et al., 2012). This may explain the excellent invasiveness of CC12 towards RAW264.7 cells and the resistance to eradication by phagocytes in the *in vivo* infection model. The CC12 strain had much more antibiotic determinants than that of CC10, which conferred resistance to macrolide, clindamycin, aminoglycoside, and tetracycline. This indicated that the environment that promoted the evolution of CC12 was complicated and included increased antibiotic pressure. Two CRISPR-Cas systems have been identified in GBS, one belonging to type II-A and the other belonging to type I-C (Pastuszka et al., 2021). In this study, CC10 only had a type II-A CRISPR-Cas system, but CC12 had both type II-A and I-C systems. The II-A system is ubiquitous, highly polymorphic, and dynamic, while the I-C system is rare and often incomplete, with seemingly little to no activity (Lier et al., 2015). In this study, the I-C system in CC12 strains was complete and seemed to be active since they had a comparable number of spacers as the II-A system. However, whether the I-C system affects CC12 virulence or antibiotic resistance features remains to be investigated further. We searched the MLST and GenBank database, and found a total of 25 ST12 strains, these strains belong to two major serotype, Ib and II (Supplementary Table S1). Hierachical cluster analysis using CRISPR sequence revealed that the CC12 strains isolated from this study were clustered into one clade along with NZ_CP036376.1, NZ_CP021862.1, NZ_CP019978.1, etc., most of them belong to serotype Ib, isolated from China, while the strains in adjacent clade belongs to serotype Ib and II, most of them were from western countries and the isolation year was former (Supplementary Table S1; Supplementary Figure S5). It infers that the CC12 strains isolated in this study might be an emerging clone. In addition, the CC12 strains possess a unique toxin/antitoxin system, the PezA/T system (also known as the epsilon/zeta system). Sedwick (2011) has found that PezT activation induces a suicide program in rapidly growing bacteria, which may promote the release of other toxins that attack their host cells or competing bacteria and protect their own. PezT has an important role in bacterial pathogenicity and is considered a virulence factor in *Streptococcus pneumonia* (Mutschler et al., 2010). Therefore, it was presumed that the PezA/T system may also be related to CC12 GBS virulence, which requires further exploration.

Although CC12 GBS is not commonly reported in pediatric invasive GBS diseases, this study revealed that the CC12 GBS was hypervirulent in animal models and clinical cases. In addition, CC12 GBS genome analysis showed that these strains possessed many antibiotic resistance determinants and multiple mobile genetic elements, inferring that the genome of CC12 GBS was variable, inclusive, and had a strong ability to capture and integrate foreign genes. It is worth noting that under antibiotic and environmental pressures, the CC12 strains have an advantage in becoming super resistant clones, similar to the notorious carbapenemase-producing *Klebsiella pneumoniae*, which has captured many plasmid-coding antibiotic resistance genes, and with worldwide prevalence, causes thorny problems in treatment and hospital infection management (Yang et al., 2021). Therefore, the prevention and control of CC12 GBS are important and should be emphasized.

This study has several limitations. First, the study was conducted at a single center, and the number of strains collected was relatively small. Further research enrolling multiple medical centers to obtain a more representative view of the Ib/CC12 GBS strain type is warranted. Second, the virulence of CC12 GBS was only verified in an infection model and the possible virulence determinants that might play a role in pathogenicity was presumed, but not verified. Further research should investigate the mechanism of Ib/CC12 GBS hypervirulence characteristics.

In conclusion, this is the first report of the clinical, microbiological, and genetic characteristics of type Ib/CC12 GBS strains isolated from pediatric patients, which cause invasive and fatal GBS disease in China. Considering the probable clonal expansion and emerging multidrug resistant isolates, ongoing monitoring for type Ib/CC12 GBS infections is warranted. Moreover, further studies focused on understanding the pathogenic mechanism and identifying treatment strategies to combat fatal GBS infection are of crucial importance in the future.

## Data Availability Statement

The original contributions presented in the study are publicly available. This data can be found at: The datasets presented in this study have been deposited in the GenBank database. The accession numbers can be found at: [GeneBank accession: JAIOKR000000000], [GeneBank accession: JAIOKS000000000], [GeneBank accession: JAIOKT000000000], [GeneBank accession: JAIOKU000000000], [GeneBank accession: JAIONE000000000] and [GeneBank accession: JAIONF000000000], respectively.

## Ethics Statement

The studies involving human participants were reviewed and approved by The Ethical Committee, Xin Hua Hospital, Shanghai Jiao Tong University School of Medicine. This is a retrospective study, and the need for signed informed consent was waived.

## Author Contributions

YL, LS, and JL: conceptualization. JL: methodology, software, writing—original draft preparation, and funding acquisition. JiaY and HG: validation. FC: formal analysis and data curation. JinY: investigation. JL and JZ: resources. LS: writing—review and editing and project administration. JL and LS: visualization. YL and LS: supervision. All authors have read and agreed to the published version of the manuscript.

## Funding

This work was supported by the Doctorial Innovation Fund of Shanghai Jiao Tong University School of Medicine [BXJ201924, 2019].

## Conflict of Interest

The authors declare that the research was conducted in the absence of any commercial or financial relationships that could be construed as a potential conflict of interest.

## Publisher’s Note

All claims expressed in this article are solely those of the authors and do not necessarily represent those of their affiliated organizations, or those of the publisher, the editors and the reviewers. Any product that may be evaluated in this article, or claim that may be made by its manufacturer, is not guaranteed or endorsed by the publisher.

## Footnotes

^1^ https://pubmlst.org/sagalactiae/
