# Supplementary material for: Emerging Fatal Ib/CC12 Hypervirulent Multiresistant Streptococcus agalactiae in Young Infants With Bloodstream Infection in China
Source: Front Microbiol. 2021 Dec 15;12:767803. doi: 10.3389/fmicb.2021.767803 (PMC8715515; doi:10.3389/fmicb.2021.767803)
Supplement: Supplementary file 1 [file Table_1.DOCX]

Supplementary Material

# Supplementary Figures and Tables

## Supplementary Figures

##
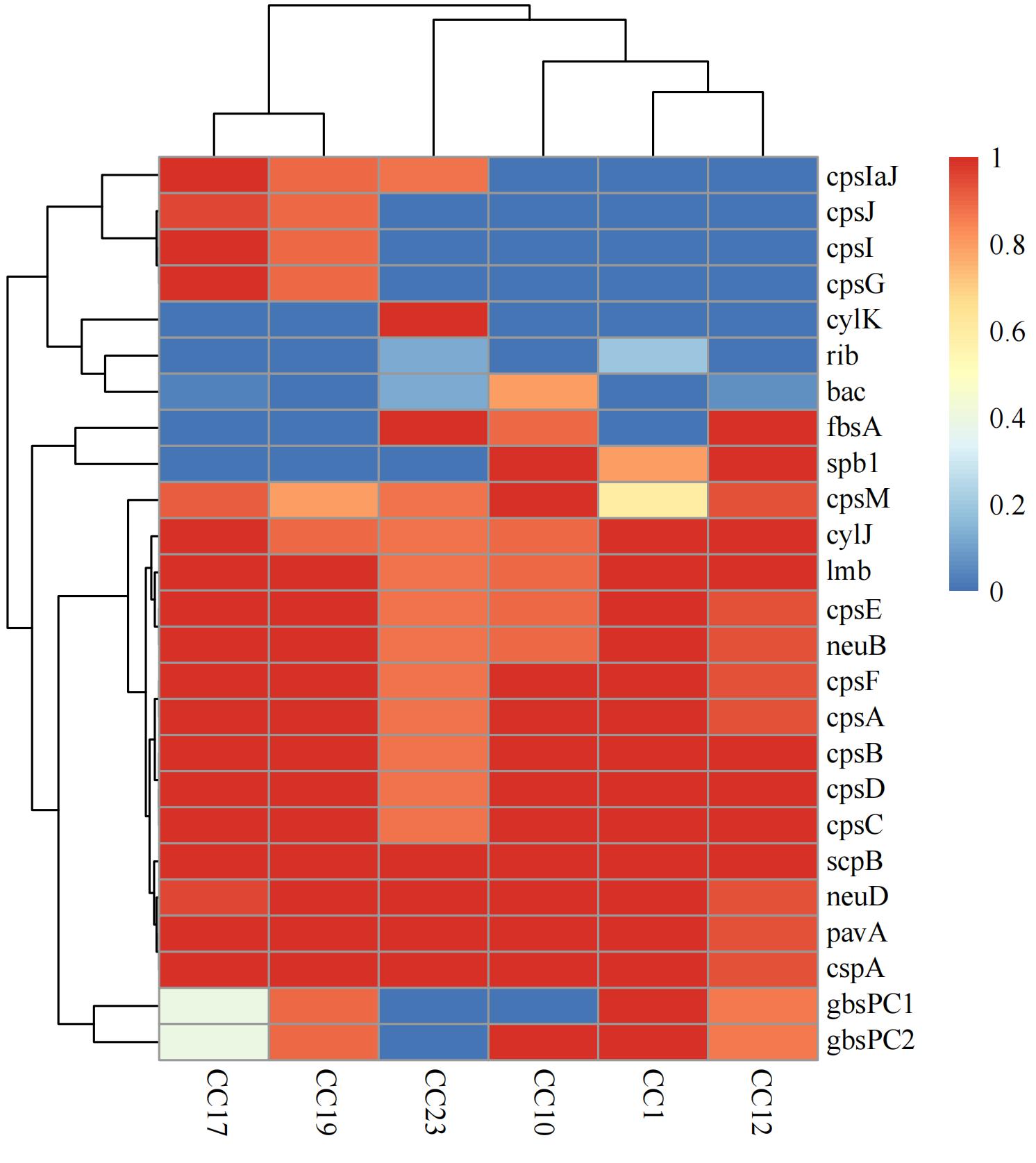


## Supplementary Figure 1. Distribution of specific virulence genes among different clonal complex (CC). The color from blue to red represent positive rate of virulence genes from 0% to 100%.

##
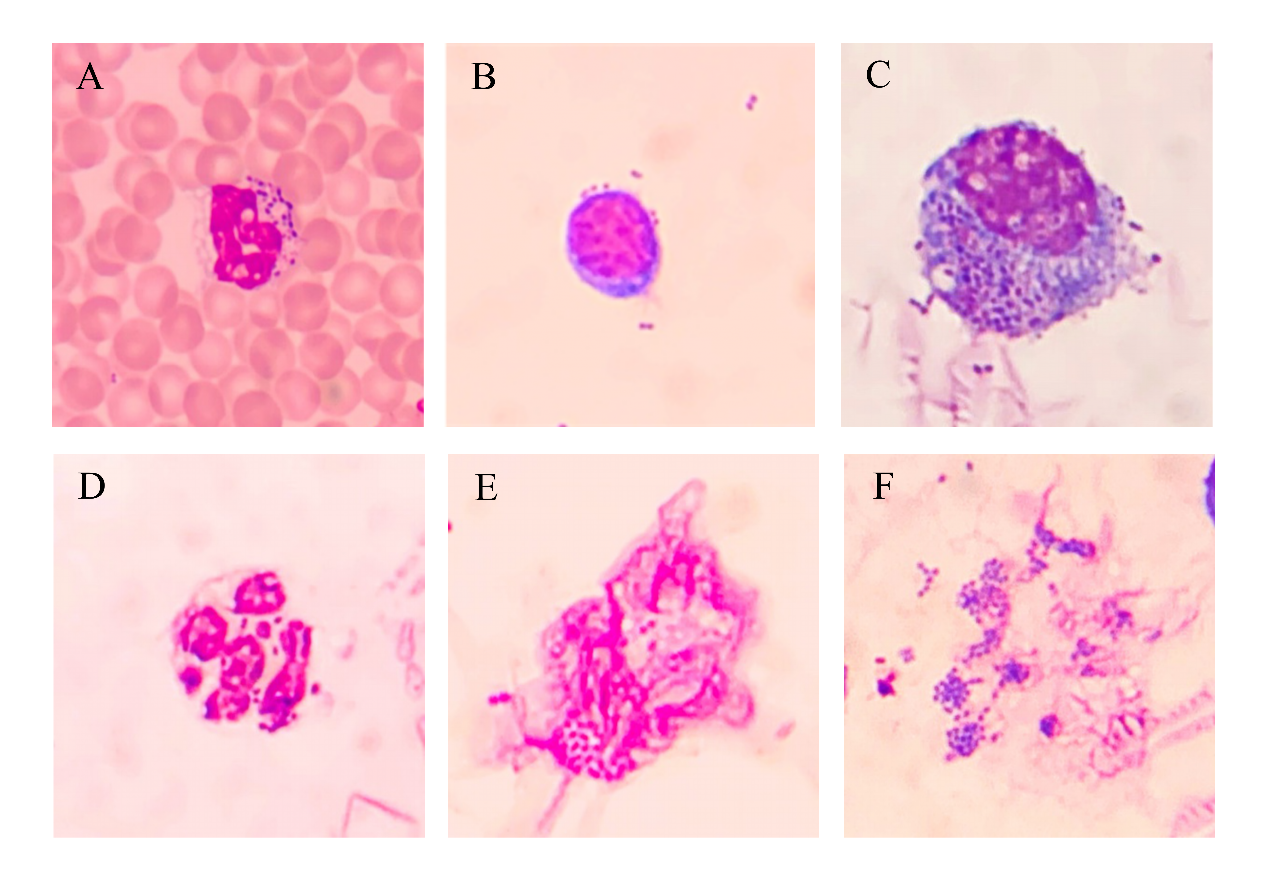


**Supplementary Figure 2.** Smear of blood and peritoneal macrophage sample extracted from clonal complex (CC) 12 infected C57BL/6, Wright's staining. A. neutrophil phagocytize bacteria in blood smear, B. lymphocyte with bacteria adhesion on surface of cell in peritoneal lavage fluid smear, C. macrophage phagocytize bacteria in peritoneal lavage fluid smear, D-F. different stage of neutrophil after phagocytize bacteria in peritoneal lavage fluid smear.


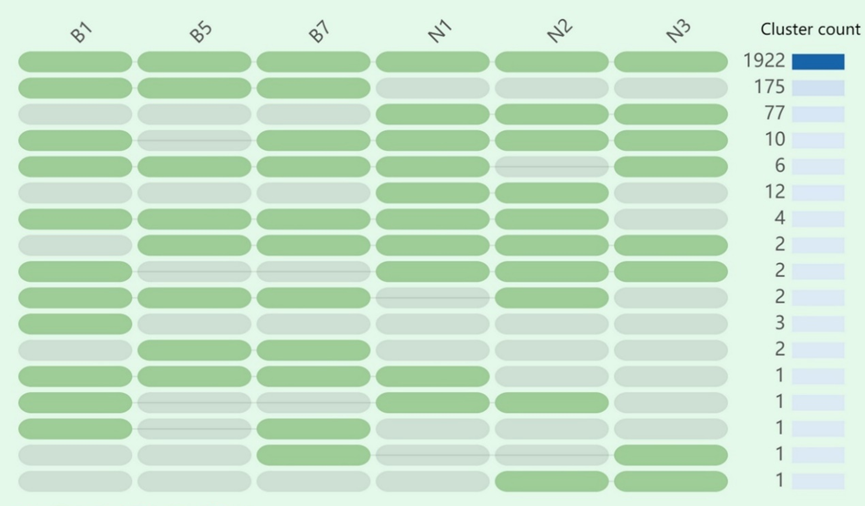


**Supplementary Figure 3.** The orthologous clusters among group B streptococcus (GBS) strains. B1, B5, B7 were clonal complex (CC) 12 strains, N1-N3 were CC10 strains. The pattern to the left indicates which strains were in the clusters, cluster count is the number of clusters shared between strains.

A

B


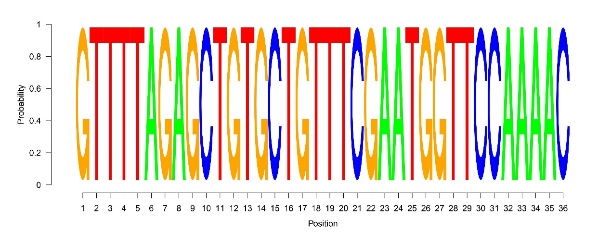

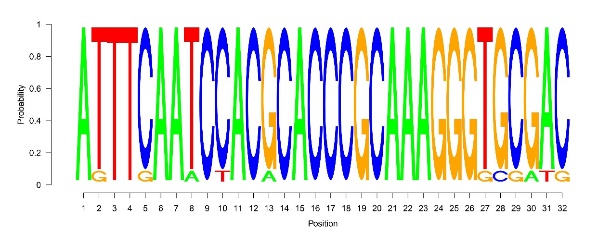


C

D


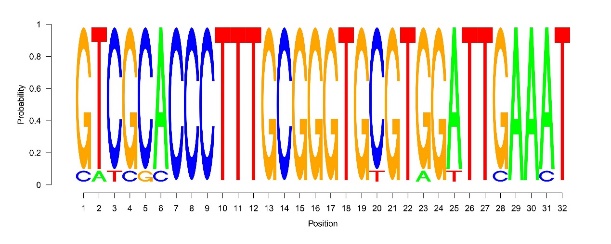

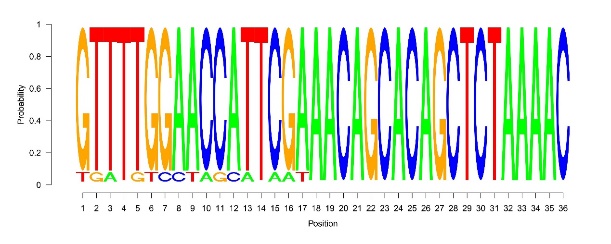


**Supplementary Figure 4.** The sequences of direct repeats (DRs) in group B streptococcus (GBS) strains. A. Ⅱ-A system of sequence type (ST) 12, B. Ⅰ-C system of ST12, C. Ⅰ-C system of ST1406, D. Ⅱ-A system of ST10


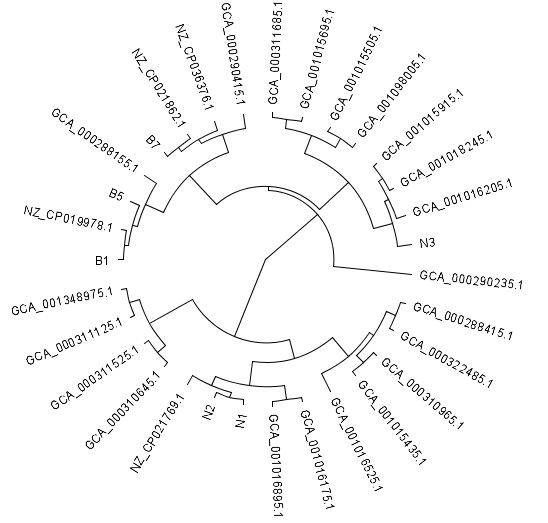


**Supplementary Figure 5.** Clustergram of CC12 strains based on CRISPR alignment. The 25 ST12 GBS isolates from GenBank and MLST database and 6 GBS strains in this study could divided into 5 clades based on CRISPR sequence using Genious 10.2.6 software. The CC12 strains in this study B1, B5, B7 were clustered in one clade.

## Supplementary Tables

**Supplementary Table1** Features of ST12 GBS strains in GenBank and MLST database

| GenBank accession | rigion | Isolated year | disease | serotypes |
| --- | --- | --- | --- | --- |
| GCA_001016895.1 | Spain | 2008 | other | II |
| GCA_001015695.1 | Italy | 2008 | other | Ib |
| GCA_001018245.1 | Denmark | 2009 | other | II |
| GCA_001016525.1 | UK | 2009 | other | II |
| GCA_001015505.1 | UK | 2009 | other | II |
| GCA_001015435.1 | UK | 2009 | other | Ib |
| GCA_001016175.1 | Portugal | 2004 | bacteraemia | III |
| GCA_001016205.1 | Portugal | 2004 | bacteraemia |  |
| GCA_001015915.1 | Portugal | 2004 | bacteraemia |  |
| GCA_001098005.1 | Bulgaria | 2009 | carrier | II |
| GCA_001348975.1 | Czech Republic | 2008 | carrier | Ib |
| GCA_000311685.1 | Unknown | Unknown | Unknown | Ib |
| GCA_000311525.1 | Unknown | Unknown | Unknown | Ib |
| GCA_000311125.1 | Unknown | Unknown | Unknown |  |
| GCA_000288155.1 | Unknown | Unknown | Unknown | Ib |
| GCA_000290235.1 | Unknown | Unknown | Unknown | Ib |
| GCA_000290415.1 | Unknown | Unknown | Unknown | II |
| GCA_000288415.1 | Unknown | Unknown | Unknown | Ib |
| GCA_000322485.1 | Unknown | Unknown | Unknown | II |
| GCA_000310645.1 | Unknown | Unknown | Unknown | Ib |
| GCA_000310965.1 | Unknown | Unknown | Unknown | Ib |
| NZ_CP036376.1 | Shanghia, China | 2017 | carrier | Ib |
| NZ_CP021862.1 | Hong Kong, China | 2008 | arthritis | III |
| NZ_CP019978.1 | Shanghia, China | 2014 | bacteraemia | Ib |
| NZ_CP021769.1 | Shenzhen, China | 2016 | carrier |  |
